# Compartment syndrome after distal biceps brachii tendon rupture in an athlete

**DOI:** 10.31744/einstein_journal/2020RC4778

**Published:** 2020-01-23

**Authors:** Carolina Ejnisman, Paulo Santoro Belangero, Carlos Vicente Andreoli, Alberto de Castro Pochini, Moises Cohen, Benno Ejnisman

**Affiliations:** 1 Faculdade de Medicina de Santo AmaroSão PauloSPBrazilFaculdade de Medicina de Santo Amaro, São Paulo, SP, Brazil.; 2 Universidade Federal de São PauloSão PauloSPBrazilUniversidade Federal de São Paulo, São Paulo, SP, Brazil.

**Keywords:** Arm injuries/surgery, Tendon injuries/surgery, Compartment syndromes, Emergencies, Athletic injuries, Rupture/surgery

## Abstract

This is a case report of a previously healthy athlete who did not use oral anticoagulant, suffered a rupture of the distal biceps brachii tendon, and evolved with arm compartment syndrome. An emergency fasciotomy and the repair of the tendon were performed. After surgery the patient had a good recovery of the paresthesia and sensibility. This complication is rare and, when reported, is usually associated with patients who use anticoagulant therapy. Due to growth of rupture of distal biceps tendon cases, physicians should be aware that this complication must be treated as an emergency.

## INTRODUCTION

In published literature there are few case reports about arm compartment syndrome, and the majority of those reported occurred after rupture of both distal and proximal biceps.^1-4^ Fung et al.,^1^ reported arm compartment syndrome after trauma in patient’s shoulder who were undergoing treatment with warfarin, fasciotomy and drainage of the injured area.

Partial rupture of brachial biceps often does not have surgical indication, however, total rupture must be repaired with surgery for reinsertion of tendon to radial tuberosity. This procedure can be done by using an access measuring about 3cm distal to elbow, or by using two accesses; one distal and the other proximal to the elbow. Immediate surgical approach is suggested, however, the injury is not considered an emergency. Although the situation is not considered an emergency, not for the rupture, such as in our case, if not treated as early as possible a number of clinical problem can occur due to the compartiment syndrome.

## CASE REPORT

A previously healthy 65-year-old patient who practice weightlifting daily was admitted to our service complaining about significant pain in his left arm 4 hours after an extension movement during a biceps curl workout in which he listened a click sound followed by extremely pain associated with deformity.

During physical exam, a distal biceps injury was diagnosed by the positive result in Hook test. The patient reported an intense swelling in his upper arm.

In the day after the episode the patient progressed with intense pain, and pins and needles in the fifth finger. After 3 hours, a worsening of parenthesis occurred, which evolved for the fourth finger.

**Figure 1:**
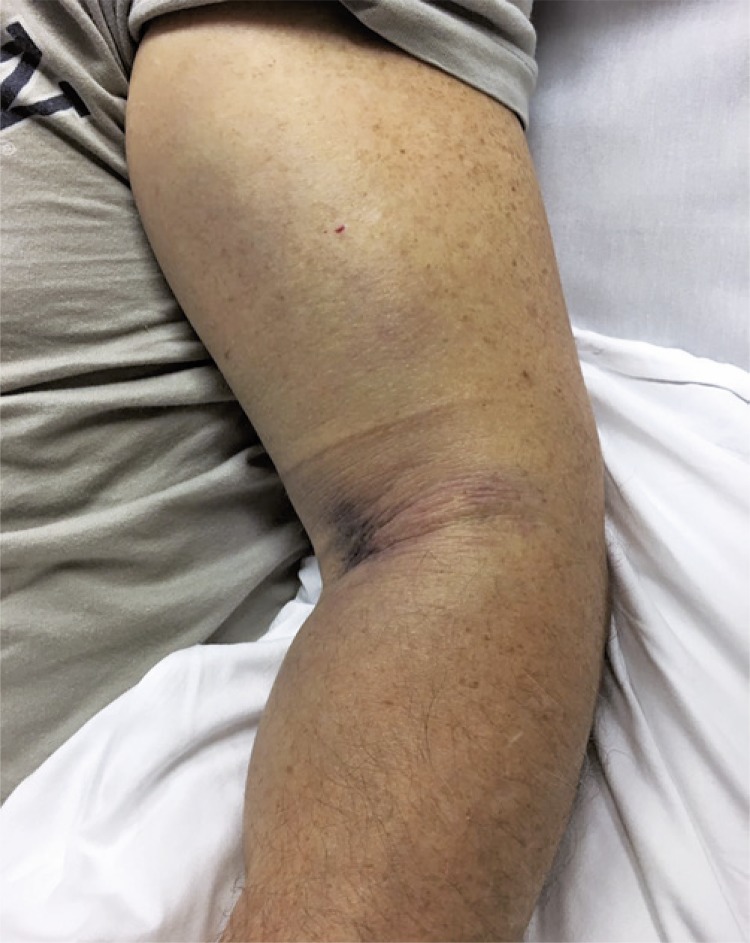
Patient’s arm with significant edema before the surgical intervention

A Doppler ultrasonography exam was conducted, but no evidence of vascular change was found, but there was a large collection of hematomas. After 1 hour, the episode evolved to worsening of paresthesia, advanced to the second and third finger with intense swelling, and a pressure feeling (Figure 1). At this time, about 12 hours after the injury episode, an immediate surgical intervention was indicated.

**Figure 2:**
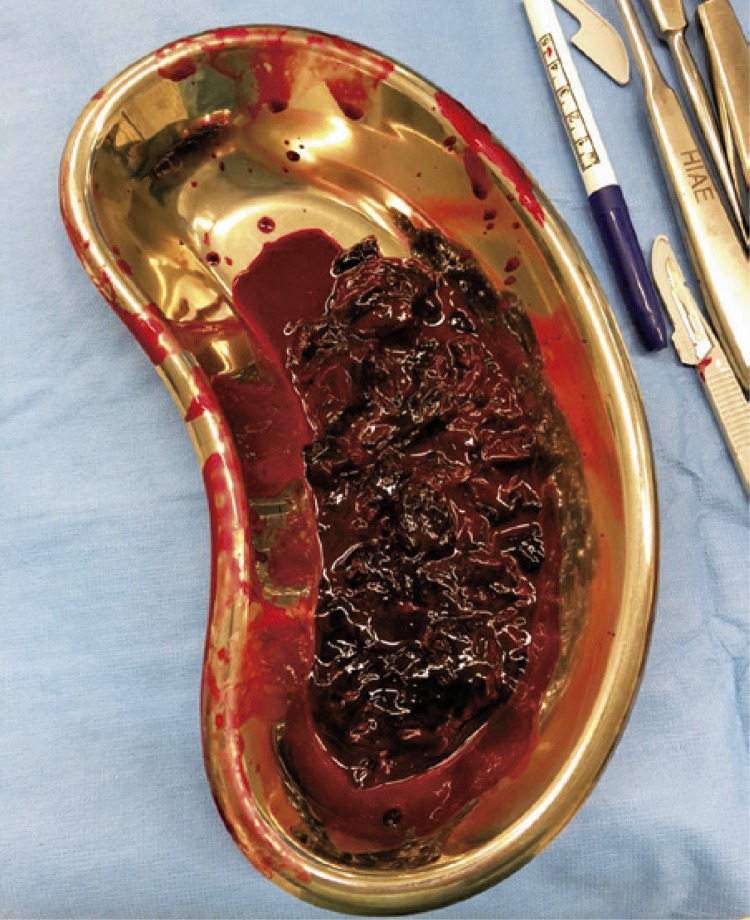
Drainage of content of the injured area during the procedure to reduce compartment pressure

**Figure 3:**
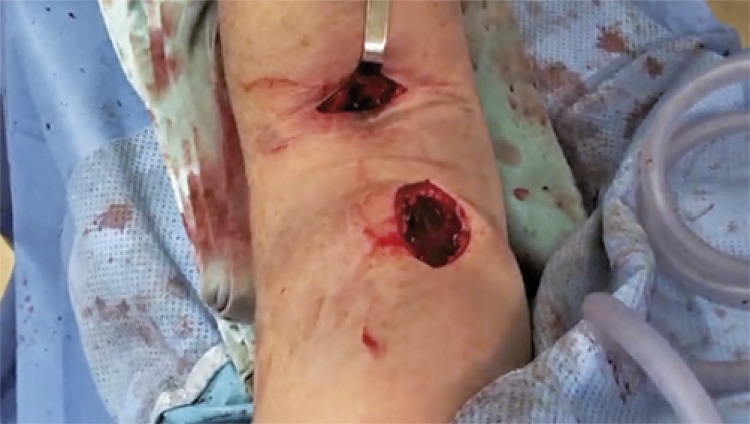
Intraoperative imaging of surgical intervention conducted by using two surgical accesses

The surgery was conducted after using a surgical access about 5cm distal to elbow to fix tendon with two absorbable anchors, and using other longitudinal surgical access proximal to elbow, the hematoma that caused the compartment syndrome was drained (Figures 2 and 3).

**Figure 4:**
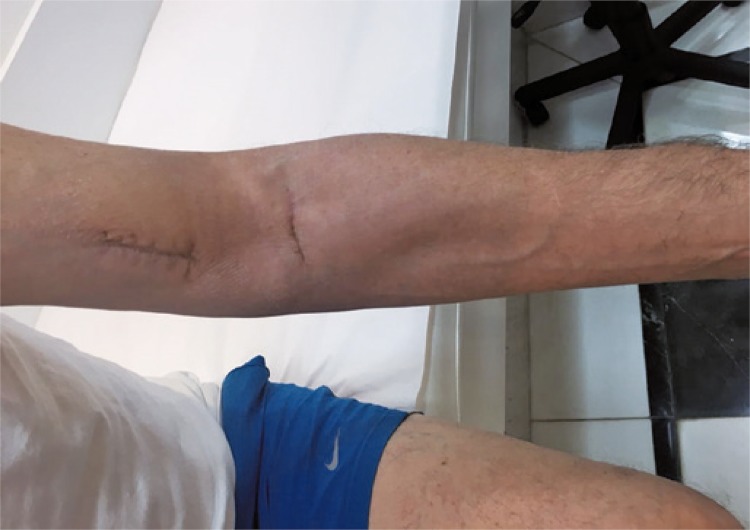
3 months follow-up and cicatrization of surgical incision

Patient had good evolution. In the follow-up assessment 3 months later with the orthopedist, after cicatrization of surgical incisions, the patient was told to return to his daily routine of exercises (Figure 4). Six months after the procedure he could return to weight training.

## DISCUSSION

Compartment syndrome represents a risk for human body extremities. This syndrome is caused by increasing of pressure inside the compartment, and constitutes a painful condition because of the hypoxy caused by tissues. In addition, compartment syndrome, if not leading to fasciotomy, may cause necrosis, loss of sensibility and, eventually, renal dysfunction and even death because of the intense rhabdomyolysis.^5^

Compartment syndrome in the arm can be considered an emergency to avoid or reduce neurological and vascular risks that can also evolve to Volkmann’s contracture.^6^

Clinically, patients may suffer the following symptoms that indicate compartment syndrome: pain, paresthesia, reduction of pulse and pallor. However, as in the case of our patient, not all clinical sings are presented, the diagnosis in our case, therefore, was evident because of the intense edema and progressive paresthesia in the fingers.

The arm presents three compartments: the anterior that contains flexure muscles of the elbow, median and ulnar nerves, which is the posterior of elbow extensors and radial nerve, and the deltoid. The anterior and the posterior compartments support large amounts of liquids, and reduces the pressure, therefore, constituting a risk for compartment syndrome,^7^ and making this complication even more uncommon. The main goal of treatment of acute compartment syndromes is decompression of affected nerves and vessels. In our case, hematic collection was found only in anterior compartment and, for this reason, a fasciotomy only of this structure was conducted.

Due to growth of biceps brachial rupture cases, possibly related to the increase of physical exercise time and intensity, as well as because of improving in clinical and radiological diagnosis,^8^ there is need for attention to the eventual complication, *i.e.*, the compartiment syndrome, especially among patients with coagulation disturbances, and also among healthy patients without any risk.

Fung et al.,^1^ reported a case of arm compartment syndrome in patient who were using warfarina. Patients using anticoagulants are more susceptible to present compartment syndrome and they constitute the most common described cases in the published literature.^9^

However, our case report is about a patient without coagulation dysfunction, who did not used medication that altered hemostasia, and who practiced physical activity, and presented the same clinical picture of compartment syndrome.

A similar case to our was reported by Grandizio et al.,^3^ after biceps distal rupture in a patient with no coagulopathy nor use of warfarina. A fasciotomy was conducted and the coagulated blood was drained, however, the incision of the drainage was 12cm and biceps tendon was repaired in another surgery two days later along with the fasciotomy incision closure. The patient of that case had a good recovery without significant neurovascular injuries.

However, Lanier et al.,^10^ in a case also similar to our including a 33-year old worker without coagulopathies, conducted a distal biceps repair without using anchors 20 days after compartment syndrome drainage. The patient evolved with 90% of arch gaining of the movement and 85% of supination strength.

In the procedure conducted in our case, different from the one performed by Grandizio et al.,^3^ and Lanier et al.,^10^ we performed two interventions in the same surgery: drainage of injured area due to compartment syndrome and repair of biceps tendon rupture. According to Maciel et al.,^11^ to conduct the repair as early as possible by single route by using suture anchors is a safety and efficient therapeutic option that present good clinical and functional results.

To our knowledge, this is the first case reported in the published literature of compartment syndrome in a patient who did not have coagulopathy, and who underwent simultaneous the fasciotomy and reinsertion of distal biceps tendon. The procedure reported was considered safe and enabled patient to return to functional and athletic activities.
