# Supplementary material for: Compartment syndrome after distal biceps brachii tendon rupture in an athlete
Source: Einstein (Sao Paulo). 2020 Jan 23;18:eRC4778. doi: 10.31744/einstein_journal/2020RC4778 (PMC6986456; doi:10.31744/einstein_journal/2020RC4778)
Supplement: Translation

# Translation: Síndrome compartimental em paciente atleta após ruptura do tendão distal do músculo bíceps do braço

**Authors:** Carolina Ejnisman, Paulo Santoro Belangero, Carlos Vicente Andreoli, Alberto de Castro Pochini, Moises Cohen, Benno Ejnisman

**Affiliations:** 1 Brasil Faculdade de Medicina de Santo Amaro, São Paulo, SP, Brasil.; 2 BrasilUniversidade Federal de São Paulo, São Paulo, SP, Brasil.

**Keywords:** Traumatismos do braço/cirurgia, Traumatismos dos tendões/cirurgia, Síndromes compartimentais, Emergências, Traumatismos em atletas, Ruptura/cirurgia

## Abstract

Relato de caso de paciente atleta, previamente hígido e que não utilizava anticoagulantes orais, com lesão do tendão distal do músculo bíceps braquial, que evoluiu com síndrome compartimental do braço. Realizaram-se fasciotomia de emergência e reparo cirúrgico do tendão, apresentando bom seguimento com recuperação da parestesia e sensibilidade. Essa complicação é bastante rara e, quando relatada, geralmente é associada a pacientes em uso de medicamentos anticoagulantes orais. Contudo, com o aumento da incidência de rupturas do tendão do músculo bíceps braquial, é preciso estar atento à tal complicação que deve ser conduzida como emergência.

## INTRODUÇÃO

Na literatura, são raros os casos de síndrome compartimental no braço, principalmente após a ruptura de bíceps, tanto distal quanto proximal.^1-4^ Fung et al.,^1^ relataram caso de síndrome compartimental após evento traumático no ombro em paciente em tratamento com varfarina, realizando-se fasciotomia e drenagem do conteúdo.

A ruptura parcial do bíceps braquial geralmente não tem indicação cirúrgica, porém, a ruptura total deve ser reparada por meio de procedimento cirúrgico, que fixe novamente o tendão à tuberosidade do rádio. Esse procedimento pode ser feito por um acesso cerca de 3cm distal ao cotovelo, ou por dois acessos, um distal e outro proximal ao cotovelo. A cirurgia tem a indicação de ser feita rapidamente, mas não é considerada caso de emergência, como foi nesse caso, não pela ruptura, mas pela síndrome compartimental associada, que pode ter diversas repercussões clínicas, se não tratada o mais cedo possível.

## RELATO DE CASO

Paciente de 65 anos, previamente hígido, praticante de musculação diariamente, compareceu à consulta com queixa de dor significativa no braço esquerdo 4 horas após realizar o movimento de extensão durante exercício de rosca direta, tendo apresentado um “estalo” seguido de muita dor, associado à deformidade.

Durante o exame físico, foi constatada a lesão do bíceps distal pelo teste de Hook positivo, tendo sido observado intenso inchaço na região anterior do braço.

Progrediu ao longo do dia com dor intensa, sendo seguida por episódios de formigamento do quinto dedo. Após 3 horas, ocorreu piora da parestesia, evoluindo para o quarto dedo.

Realizou exame de Doppler ultrassonografia, que não evidenciava alteração vascular, mas com grande coleção de hematomas.

Após 1 hora, evoluiu com piora da parestesia, avançando para o segundo e terceiro dedo, tendo intenso inchaço e sensação de pressão (Figura 1). Neste momento, cerca de 12 horas após a lesão inicial, foi indicada intervenção cirúrgica imediata.

**Figura 1:** Braço do paciente apresentando edema significativo antes da intervenção cirúrgica

Neste caso, a cirurgia foi realizada por meio de acesso cerca de 5cm distal ao cotovelo, para fixar o tendão com duas ancoras absorvíveis, e com outro acesso longitudinal, proximal ao cotovelo, com a finalidade de drenar o hematoma que ocasionava a síndrome compartimental (Figuras 2 e 3).

**Figura 2:** Conteúdo drenado durante o procedimento para reduzir a pressão do compartimento

**Figura 3:** Imagem intraoperatória da intervenção cirúrgica conduzida com dois acessos

Paciente evoluiu bem. Em avaliação com o ortopedista, 3 meses depois, já apresentava pleno retorno às atividades cotidianas e cicatrização das incisões cirúrgicas (Figura 4). Seis meses após o procedimento, orientado em consulta de retorno, retomou as práticas esportivas.

**Figura 4:** Paciente com 3 meses de seguimento e cicatrização das incisões cirúrgicas

## DISCUSSÃO

A síndrome compartimental representa risco às extremidades do corpo humano. É causada pelo aumento da pressão dentro de compartimento, sendo condição dolorosa, em decorrência da hipóxia causada aos tecidos. Pode causar, se não realizar-se a fasciotomia, necrose, perda da sensibilidade e, eventualmente, disfunção renal e até morte decorrentes de intensa rabdomiólise.^5^

A síndrome compartimental no braço deve ser conduzida como emergência para evitar ou minimizar riscos neurológicos e vasculares, que também podem evoluir para contratura de Volkmann.^6^

Clinicamente, o paciente pode sofrer os seguintes sintomas que indicam a síndrome compartimental: dor, parestesia, diminuição do pulso e palidez. Entretanto, como no caso do paciente, nem todos sinais clínicos se apresentaram, o diagnóstico, então, foi valorizado devido ao edema intenso e à parestesia progressiva dos dedos.

O braço apresenta três compartimentos: o anterior, que contém os músculos flexores do cotovelo, os nervos ulnar e mediano; o posterior, dos extensores do cotovelo e o nervo radial; e o deltoide. Os compartimentos anterior e o posterior suportam grandes volumes de líquidos, limitando a pressão e, assim, os riscos de síndrome compartimental,^7^ tornando essa complicação ainda mais rara. O objetivo principal do tratamento das síndromes compartimentais agudas é a descompressão de todos os nervos e vasos afetados. No caso apresentado, a coleção hemática apresentava-se somente no compartimento anterior e, por isso, realizou-se a fasciotomia apenas desta estrutura.

Com o aumento dos casos de ruptura do bíceps braquial, provavelmente relacionado ao aumento da prática de atividades física e à sua intensidade, bem como melhora do diagnóstico clínico e radiológico,^8^ é preciso estar atento à essa eventual complicação, a síndrome compartimental, especialmente em pacientes com distúrbios de coagulação, mas também em pacientes hígidos sem qualquer indício de alterações.

O caso de Fung et al.,^1^ relatou síndrome compartimental no braço em paciente em uso de varfarina. Pacientes em uso de anticoagulantes são mais suscetíveis a apresentar síndrome compartimental e apresentam mais casos descritos na literatura.^9^

Esse caso, contudo, apresenta paciente sem disfunção de coagulação ou uso de medicamento que alterasse a hemostasia, praticante de atividade física, que apresentou esse mesmo quadro de síndrome compartimental.

Caso muito semelhante ao nosso foi relatado por Grandizio et al.,^3^ após ruptura do bíceps distal, em paciente sem coagulopatia nem uso de varfarina. Foram realizadas fasciotomia e drenagem do sangue coagulado, mas a incisão para drenagem era de 12cm e o reparo do tendão do bíceps foi feito em outra cirurgia, após 2 dias, em conjunto com o fechamento da incisão da fasciotomia. Apresentou boa recuperação, sem lesões neurovasculares significativas.

Já Lanier et al.,^10^ com um caso também semelhante ao nosso em trabalhador de 33 anos sem coagulopatias, realizaram o reparo do bíceps distal com uso de duas âncoras após 20 dias da drenagem da síndrome compartimental. O paciente evoluiu com 90% do ganho de arco de movimento e 85% da força de supinação.

No procedimento conduzido nesse caso, diferente do realizado por Grandizio et al.,^3^ e Lanier et al.,^10^ foram feitos os dois procedimentos na mesma intervenção cirúrgica: drenagem do conteúdo proporcionou a síndrome compartimental e reparo do tendão do bíceps. Segundo Maciel et al.,^11^ o reparo o mais precoce possível por via única com uso de âncoras de sutura mostra-se opção terapêutica segura e eficaz com bons resultados clínicos e funcionais.

Este é o único caso relatado na literatura de síndrome compartimental em paciente sem coagulopatia realizando simultanemente a fasciotomia e a reinserção do tendão do bíceps distal. O procedimento relatado demonstrou ser seguro e possibilitar bom retorno funcional e atlético do paciente.
